# System Performance and User Feedback Regarding Wearable Bioimpedance System for Multi-Site Knee Tissue Monitoring: Free-Living Pilot Study With Healthy Adults

**DOI:** 10.3389/felec.2022.824981

**Published:** 2022-04-21

**Authors:** Shelby Critcher, Todd J. Freeborn

**Affiliations:** Department of Electrical and Computer Engineering, the University of Alabama, Tuscaloosa, AL, United States

**Keywords:** wearable, bioimpedance, multi-modal, knee, multi-site

## Abstract

Knee-focused wearable devices have the potential to support personalized rehabilitation therapies by monitoring localized tissue alterations related to activities that reduce functional symptoms and pain. However, supporting these applications requires reported data to be reliable and accurate which can be challenging in the unsupervised free-living conditions that wearable devices are deployed. This pilot study has assessed a knee-focused wearable sensor system to quantify 1) system performance (operation, rates of data artifacts, environment impacts) to estimate realistic targets for reliable data with this system and 2) user experiences (comfort, fit, usability) to help inform future designs to increase usability and adoption of knee-focused wearables. Study data was collected from five healthy adult participants over 2 days, with 84.5 and 35.9% of artifact free data for longitudinal and transverse electrode configurations. Small to moderate positive correlations were also identified between changes in resistance, temperature, and humidity with respect to acceleration to highlight how this system can be used to explore relationships between knee tissues and environmental/activity context.

## 1 INTRODUCTION

Emerging technology and investigation into data driven healthcare has resulted in wearable devices becoming increasingly popular for health-driven applications. This language, wearable devices (or wearables), refers to electronic sensing circuitry integrated into body-worn form factors (e.g., clothing, jewelry, shoes). Wearables collect, process, and report data related to the person or environment in which they are worn. A major aim of these devices is to provide near-continuous physiological and health data during free-living (Patel et al., 2012; Düking et al., 2016) that can inform health-related decision making. These devices can integrate one or more sensing technologies (e.g., accelerometry, photoplethysmography (PPG), electrocardiography (ECG), electromyography (EMG), bioimpedance spectroscopy (BIS), acoustics, temperature, etc.) and can be on a variety of different body sites including the wrist, ear, face/neck, head, hands, torso, and legs. As an example of generated health-data, wrist and ear-worn devices have been utilized in previous studies to collect blood-pressure, heart-rate, and oxygen levels (Poh et al., 2010; Lee et al., 2011; Budidha and Kyriacou, 2014; Thomas et al., 2015). Beyond research, wearable devices are emerging as a significant market with a size of $36 billion USD in 2019 with projections to increase approximately 16% annually until 2027 (Research, 2020). The potential for advancing health-focused applications and economic opportunities continue to motivate research and development efforts focused on wearable devices.

There are two broad categories of wearable devices: 1) those requiring direct tissue contact and 2) those without direct tissue contact. Consider inertial measurement sensors that characterize acceleration and rotation, these sensors can be worn on a body site without contacting the tissue while generating data for applications such as monitoring respiration rate (Liu et al., 2011) and motion recognition (Yeoh et al., 2008). These sensors capture an outcome of a physiological process (in the case of respiration muscle contraction/relaxation that changes torso geometry) but not direct details of the internal physiological processes. In contrast, sensing technologies such as PPG, ECG, or BIS which require direct contact to the tissue, capture specific details about processes internal to the tissue. The selection of sensing modalities for a wearable device is driven by the target health-aim and the constraints of the body site of interest.

One sensing modality that is being increasingly investigated to characterize localized tissues is bioimpedance spectroscopy. This modality measures the passive electrical impedance of a biological tissue which is dependent on the tissue type, fluid content, structure, and geometry. BIS has been investigated for applications related to knee-joint health (Neves et al., 2009; Hersek et al., 2016), neuro-muscular disorders (Sanchez and Rutkove, 2017), muscle injuries (Nescolarde et al., 2013; Nescolarde et al., 2020), skeletal muscle fatigue (Fu and Freeborn, 2018; Freeborn et al., 2020), breast cancer detection (Mansouri et al., 2020), fluid shifts (Fenech and Jaffrin, 2004), blood pressure monitoring (Wang et al., 2020; Sanchez-Perez et al., 2022), and respiration rate monitoring (Aqueveque et al., 2020; Blanco-Almazan et al., 2020; Heydari et al., 2020; Pavlin et al., 2021). Wearable BIS devices, specifically for the knee, could support monitoring of joint-fluids (e.g. swelling), soft-tissue architecture (e.g. skeletal muscle, tendons), and joint spacing in support of both rehabilitation activities and tracking disease progression (e.g., knee osteoarthritis). With regards to knee osteoarthritis, knee-focused wearables could also support rehabilitation therapies by monitoring tissue alterations related to activities that reduce both functional symptoms and pain. The addition of BIS provides the opportunity for direct tissue insight to be included in rehabilitation tracking, expanding beyond current approaches that use only motion sensing (Li et al., 2017; Naeemabadi et al., 2020).

While there are a range of portable instrumentation available for BIS that support laboratory and clinical work, wearable options to collect data in free-living environments are limited to research prototypes (Hersek et al., 2016; Rossi et al., 2017; Teague et al., 2020; Dheman et al., 2021) which are often only validated in controlled laboratory settings. Data collected in unsupervised free-living environments can have errors introduced by motion artifacts, electrode disconnect events, electrode aging, cabling damage/disconnects, and electronics/sensor damage. These sources of errors can be eliminated or drastically reduced under supervision but knowledge on how these errors effect the functionality and data interpretation from a wearable BIS device in a free-living environment is currently unknown. Recent efforts have produced a prototype system to generate multi-modal (BIS, acoustic, inertial, temperature) in support of joint health applications (Teague et al., 2020). However, a noted limitation of the design by Teague et al. was the form factor required precise placement of multiple bulky components by the user. This could be revised to improve comfort and conformity between device and user. This need to advance designs for improved comfort/repeated usability and evaluate the quantity of reliable data generated in unsupervised free-living environments motivates this effort.

The aim of this study is to evaluate the performance of a knee-focused wearable system for multi-modal data collection (BIS, temperature, humidity, acceleration) during unsupervised use in free-living environments. This included identifying the amount of reliable data generated by 5 healthy adult participants wearing the system during across 2 days during their unsupervised daily living. Additionally, user experiences from the study participants are analyzed to determine the comfort/usability of this system to help inform revisions for future designs. The following sections outline the knee-focused wearable system, the data collection process, methods to identify artifact-free data, and samples of relationships between contextual and tissue impedance data to serve as sites of future research with knee-focused wearables.

## 2 WEARABLE SYSTEM DESIGN

**FIGURE 1 ∣:**
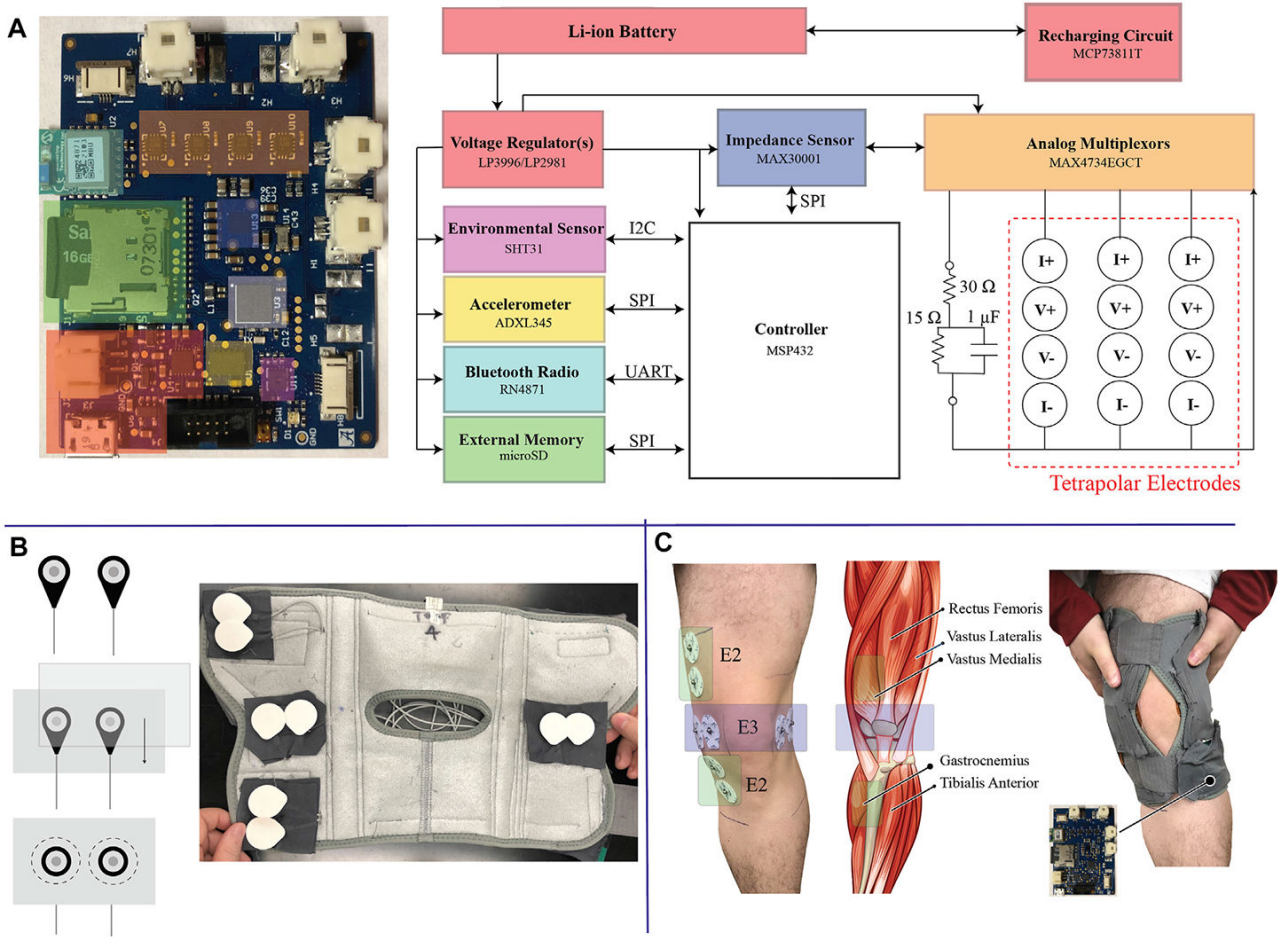
**(A)** High-level block diagram of the electronics of the wearable sensor system, **(B)** electrode integration into the commercial brace, and **(C)** illustration of knee tissue locations that are measured by the wearable system after fitting to a participants body.

The wearable design used to collect knee tissue and contextual measurements (e.g., activity, temperature, and humidity) integrated an electronic sensing system realized as a rigid printed circuit board into a commercially available textile knee brace (Thermal Vent Open Knee Wrap Stabilizer, Swede-O). The brace was modified to include cabling and electrodes for the electronics/tissue interface. The system electronics and modified knee-brace are shown in Figure 1. A commercial knee brace was chosen because these products are widely used in the treatment of knee injuries and diseases impacting the knee (Richards et al., 2005; Robert-Lachaine et al., 2020). This wearable choice aims to leverage familiarity with this type of textile to reduce barriers to setup and adoption by participants and to take advantage of fabric and sizing choices for comfort and usability. The selected knee-brace utilized a knee-wrap design and not a knee-sleeve design to support adults with different abilities (e.g., knee range of motion, bending) to install and wear on their own. A knee-wrap design without obtrusive mechanical bracing/inserts was selected to improve comfort for users.

### 2.1 Electronic Sensing System

The electronic sensing system was designed to collect and store tissue impedance data from multiple knee locations and activity/environmental context data. A high-level block diagram of the system and the fabricated printed circuit board (PCB) is shown in Figure 1A which has the following sub-systems:

- Power Management: Regulate the lithium ion battery voltage to necessary supply voltages (1.8 V, 3.3 V) and provide on-board re-charging functionality;
- Controller: System controller (MSP432 from Texas Instruments) to configure on-board sensors, coordinate data collection/storage/reporting tasks, and system monitoring (e.g., battery voltage monitoring, bioimpedance functionality);
- Bioimpedance Sensing: Bioimpedance sensing (MAX30001 from Maxim Integrated) with supporting analog multiplexors (MAX4734 from Maxim Integrated) to measure electrical impedance from up to 3 on-body locations and 1 on-board test-impedance;
- Context Sensing: 3-axis acceleration sensing (ADXL345 from Analog Devices) to quantify movement of the wearable/knee and temperature & humidity (SHT31 from Senserion) to quantify environmental conditions;
- Data Storage/Communications: On-board microSD card for data storage and Bluetooth transceiver (RN4871 from Microchip) for wireless communications.

Each sub-system is given a unique block color in Figure 1A that corresponds to its location on the PCB. This PCB is a 6-layer design that measures 5.7 cm by 4.5 cm (25.65 cm^2^).

The MAX30001, an integrated circuit with analog front end circuitry designed for bioimpedance and biopotential measurements, provides a single chip BIS solution. This IC generates/applies a square-wave excitation signal (with 11 user selectable frequencies ranging from 125 Hz to 128 kHz) to the tissue/device under study. The voltage monitored by the MAX30001 is high-pass filtered, demodulated to DC, anti-alias filtered, amplified, and converted to a digital representation using an on-board ΣΔ analog-to-digital converter (ADC) for further digital processing (filter/decimation). Demonstration of MAX30001 functionality and application for collecting localized bioimpedance has been demonstrated in recent works (Critcher and Freeborn, 2020, Critcher and Freeborn, 2021a; Critcher and Freeborn, 2021b). It was selected for this design because it is an off-the-shelf IC with multi-frequency functionality requiring no addition front-end circuitry to collect tetrapolar impedance measurements. It is also available in a ball-grid array (BGA) package with 8 mm^2^ size, supporting its integration into a wearable system where a small form-factor is desired. It is important to note that the MAX30001 is just one of many options to measure tissue bioimpedance and that there is significant active research exploring different measurement schemes, their realizations, and their sources of error. Readers interested in these topics are directed to the comprehensive review by Naranjo-Hernández et al. (2019) for further details.

### 2.2 Multi-Site Tissue Interface

The MAX30001 sensor is designed to collect measurements from a single electrode configuration, limiting measurements of tissues to a single site in its default configuration. To expand the functionality to collect measurements from multiple locations analog multiplexers were integrated to switch the current/voltage signals to one of four tetrapolar configurations. Each tetrapolar configuration has four associated signals. Two correspond to the applied current excitation (I+, I−) and two correspond to the voltage sensing (V+, V−). The system controller coordinates selection of the multiplexor channels for appropriate routing of the signals to the desired test location (e.g., cabling that is interfaced to a tissue site or on-board test impedance).

**FIGURE 2 ∣:**
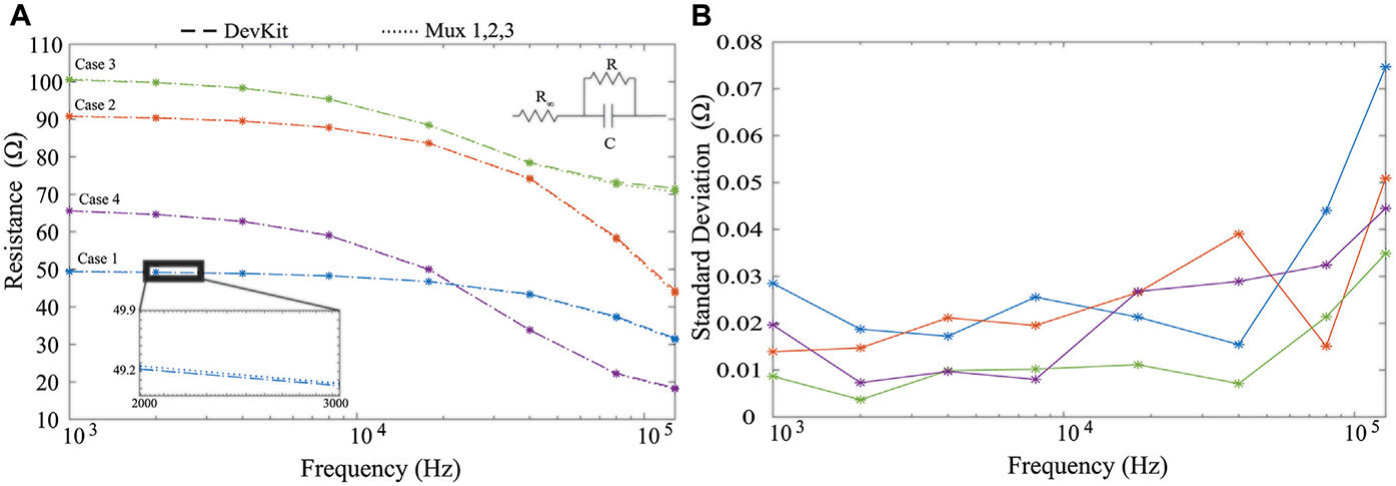
MAX30001 development kit (dashed) and wearable system (hatched) **(A)** resistance measurements from four cases of 2*R*-1*C* models and **(B)** standard deviation of measurements across all analog multiplexer configurations.

To quantify the effect of the analog multiplexers and to ensure the addition of the multiplexers did not degrade measurement quality, multi-frequency measurements were collected and reported from 4 2*R*-1*C* models (realized with discrete values to emulate localized tissue impedances). Measurements obtained from a MAX30001 development kit were used as the reference measurement and compared to measurements collected from the designed sensor system (Critcher and Freeborn, 2020). The 2*R*-1*C* values for each of the measured case were [*R*_∞_, *R*, *C*] = [20 Ω, 30 Ω, 0.047 *μ*F] [20 Ω, 71.5 Ω, 0.022 *μ*F] [71.5 Ω, 30 Ω, 0.22 *μ*F] [16 Ω, 51 Ω, 0.10 *μ*F] referred to as cases 1–4, respectively. The resistance measured by the MAX30001 development kit and the sensing system PCB for 2*R*-1*C* realization are given in Figure 2A as dashed and dotted lines, respectively. Measurements from the development kit and wearable show little visual deviation, which is further highlighted in the subset of Figure 2A. The resistance standard deviation across the measurements of each realization using the 3 multiplexor configurations is shown in Figure 2B, with less than 0.08 Ω across the measured frequency band. This supports that the multiplexers do not degrade resistance measurements collected by the MAX30001. Further assessments of the MAX30001 accuracy and precision have been reported for interested readers (Critcher and Freeborn, 2021a). While deviations up to 10% were reported for MAX30001 measurements of complex impedance in ranges expected of localized tissues, the reported precision was approximately 0.2 Ω. This level of precision supports the use of this IC for tracking relative changes which is the focus in this wearable.

To interface the system PCB to target tissue locations, flexible cabling is integrated throughout the brace. This cabling connects to the PCB using locking connectors and interfaces to adhesive Ag/AgCl electrodes using a standard electrode snap connector. The locking connectors minimize the chance of cable disconnect events occurring as a result of dynamic movement. The snap-connectors of the cabling system were placed in fixed locations of the brace to collect both longitudinal and transverse bioimpedance measurements of the knee using 2 tetrapolar configurations (requiring a total of eight electrodes in the brace). A sample of this setup with installed Ag/AgCl electrodes is given in Figure 1B. The connectors were secured into the brace by first stitching them between fabric sheets that were then sewn into the brace, also highlighted in Figure 1B. This helped maintain uniformity between electrode spacing during modifications of the brace and simplified rework for replacing cables/electrodes. The electrode position was pre-determined through the placement of these fabric sheets, reducing the effort needed by participants to configure and use the device rather than having them individually place the electrodes based on anatomical features.

The placement of electrodes targeted both longitudinal (referred to as the E2 electrode set) and transverse (referred to as the E3 electrodes) measurements of the knee tissues. The specific locations of these electrodes on a participants knee tissues (without the brace) and the underlying anatomy of the leg/knee are shown in Figure 1C. Longitudinal measurements are expected to capture bioimpedance measurements associated with skeletal muscle above/below the knee joint, knee joint tissue, and fluids in these regions while the transverse measurements are expected to be associated with knee joint geometry and fluid. This pairing provides an opportunity to identify differences in bioimpedance measurement alterations localized to the knee joint (E3) and knee joint/musculature (E2). This also allows for the investigation into whether multiple localized measurements are necessary to capture tissue dynamics related to different mechanisms (e.g., muscle activation, swelling, synovial fluid alterations, joint space alterations).

An on-board 2*R* – 1*C* model realized using discrete components is included for self-testing towards identifying and isolating functionality problems with the MAX30001 or tissue interfacing. This 2*R* – 1*C* model, with topology given in Figure 1A with component values *R*_∞_ = 30 Ω, *R*1 = 15 Ω, and *C* = 1 *μ*F, emulates a frequency dependent impedance with characteristics similar to biological tissues. Measurements of this model supports run-time validation that resistance and reactance data reported by the MAX30001 is within expected tolerances (to be used during review of data to identify degraded data that could have resulted from operational errors).

### 2.3 System Operation

**FIGURE 3 ∣:**
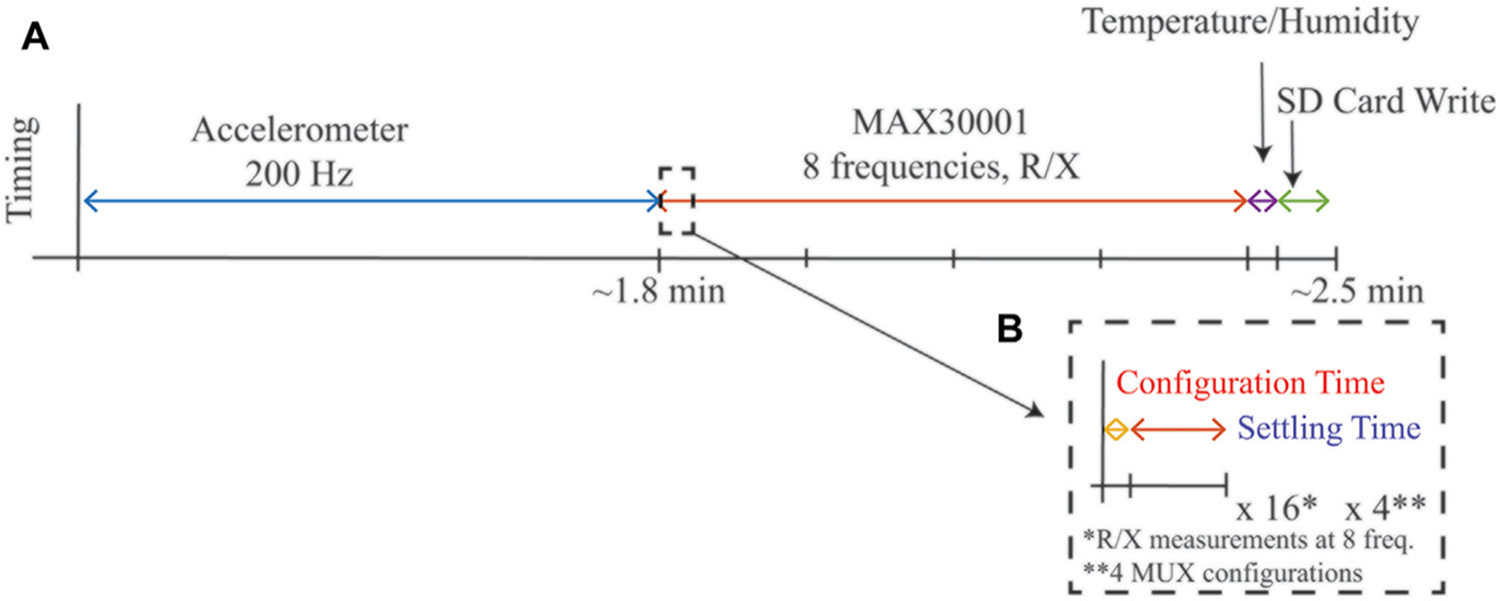
**(A)** Timing of a complete cycle of measurements from the wearable system during run-time and **(B)** highlight of configuration and settling time required for MAX30001 sensor for single frequency measurement.

Upon power-up, the MSP432 controller initialized all necessary internal hardware peripherals (e.g., timers, ADCs, GPIO, SPI, I2C, UART) and configured on-board sensors (MAX30001, ADXL345, SHT31) for data collection. After initialization, the data collection sequence was: 1) collect 3-axis accelerometer data at 200 Hz until an on-chip flash memory bank filled (approximately 1.8 min of data collection), 2) collect resistance and reactance data at 1 kHz, 2 kHz, 4 kHz, 8 kHz, 18 kHz, 40 kHz, 80 kHz, and 128 kHz from all four tetrapolar configurations, 3) collect temperature, humidity, and battery voltage measurement, and 4) write all data to the on-board micorSD card as an ascii-file (.csv format). This sequence was repeated continuously after initialization while the system was powered. The timing of this sequence, which required approximately 2.5 min, is given in Figure 3A. A limitation of the MAX30001 is that it requires reconfiguration to measure each discrete frequency after which a settling period is required before valid data is available. Figure 3B outlines the configuration vs settling time required for each change in frequency to highlight that the settling time limits the rate at which multi-frequency data can be collected with this IC. For a more comprehensive description of the MAX30001 operation and these settling times, interested readers are directed to recent demonstrations of the MAX30001 functionality (Critcher and Freeborn, 2021a). For this pilot study, important settings of the MAX30001 included utilizing its low power mode, an 80 V/V instrumentation amplifier gain, 62.5 Hz sample reporting, and an 8 *μ*A excitation current with digital filtering disabled. Note, this excitation current is compliant with IEC 60601 requirements for basic safety of electrical equipment used in medical practice. Each discrete resistance or reactance measurement that was saved for post-processing is the mean of eight measurements collected at the 62.5 Hz sample rate, representing the average over approximately 0.38 s.

## 3 PILOT STUDY OF HEALTHY ADULTS DURING FREE-LIVING CONDITIONS

A small pilot study was completed to evaluate the performance of the wearable system, alterations in knee tissue bioimpedance during free-living, and to collect user feedback. Five healthy-adult participants (1 Man, 4 Women, 23.8 years average age) were recruited, trained, and wore the knee brace across 2 days of unsupervised free-living. Prior to their participation, participants provided their written informed consent. All participants reported no history of knee pain and no previous knee injuries. This research and its activities were approved by The University of Alabama’s Institutional Review Board (UA IRB-18-013-ME).

**FIGURE 4 ∣:**
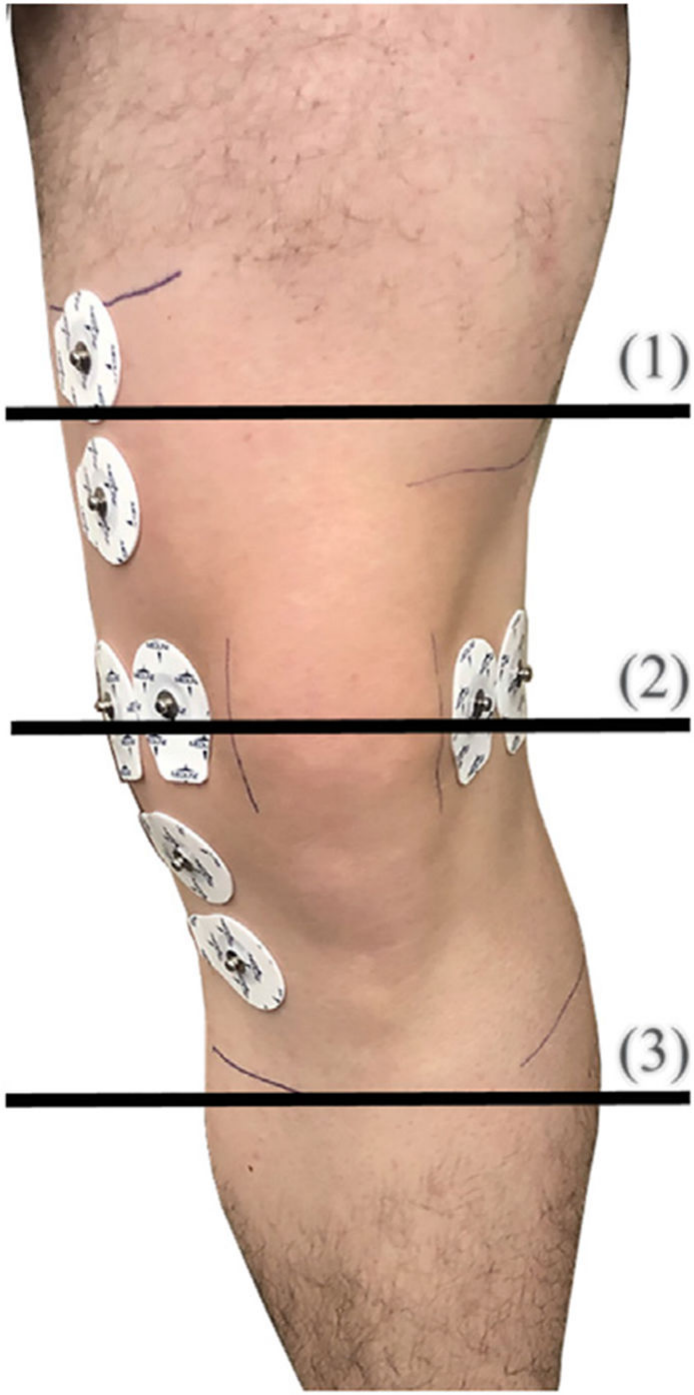
Circumference measurements around the knee at three locations.

After recruitment and consent, participants individual knee dimensions were measured at three locations to identify the appropriate brace size to be used during the trial. Leg circumference (rounded to the nearest 0.25 inches) were measured using a cloth tape measure at the locations shown in Figure 4. The measurements of all five participants are detailed in Table 1 with M1, M2, and M3 corresponding to the locations 1–3, respectively, shown in Figure 4. Based on these dimensions, participants were assigned sizes from small (S/M) to 3XL (this sizing is specific to the Swede-O Thermal Vent Open Knee Wrap Stabilizer).

### 3.1 Participant Training

Participants were trained by study personnel on setting up and using the wearable system. This included steps to connect the battery to the system, recharging the battery, cleaning the knee sites, placement of Ag/AgCl electrodes in the wearable, and placement/tightening of the wearable system on the knee. Participants first watched a step-by-step demonstration by study personnel. Next, they were required to complete each step under supervision by the study personnel with an opportunity to ask questions. Each training step was repeated until participants were able to successfully complete them without directions or corrections from study personnel. This training required approximately 1 h for each session after which participants were supplied with a kit that included the wearable system, electrodes, recharging equipment, isopropyl alcohol wipes, and an instruction manual with text and photographic instructions to supplement their in-person training.

For both days of the pilot data-collection participants were instructed to: 1) setup the brace as trained each morning (within 30 min of waking), 2) wear the brace for 10 or more hours during their regular daily activities, 3) remove the brace and electrodes in the evening and, 4) re-charge the system for use the following day. After 2 days, participants returned the kits back to the study personnel. The collected data (stored as ascii-text files) were downloaded from the microSD cards for decoding and post-processing in MATLAB.

### 3.2 On-Board MAX30001 Self-Testing

**FIGURE 5 ∣:**
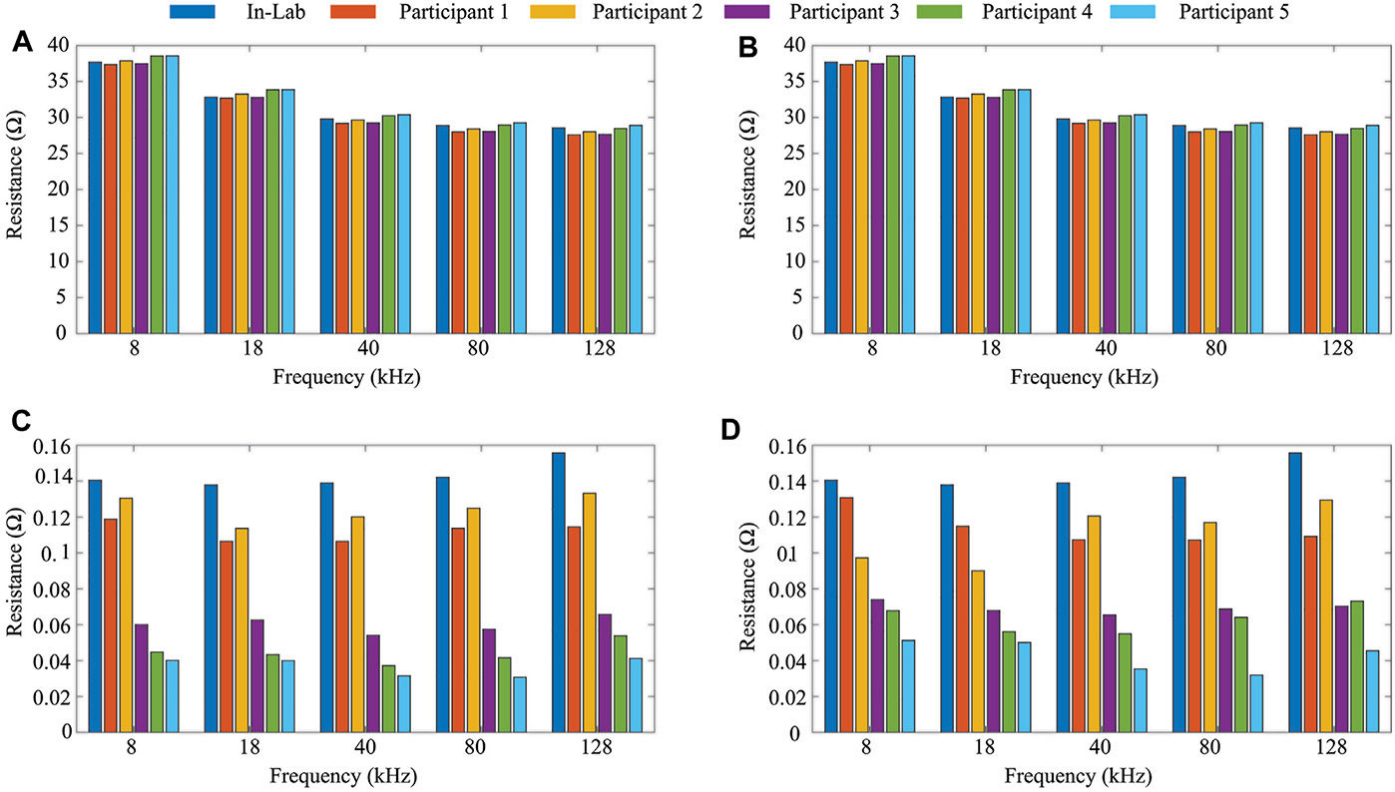
Average **(A,B)** and standard deviation **(C,D)** resistance (8–128 kHz) of on-board 2*R*-1*C* model for days 1 and 2 of data collection from study participants.

To confirm operation of the MAX30001 over the 2-day pilot study, resistance and reactance measurements of the on-board 2*R*-1*C* model (*R*_∞_ = 30 Ω, *R*1 = 15 Ω, and *C* = 1 *μ*F shown in Figure 1A) were reviewed to identify possible time-periods of sensor failure (e.g., periods where reported sensor data exceeded the expected value by ± 10% or more). Resistances measured at 8–128 kHz at similar timepoints across participants 1-5 were within a range of < 1.51 Ω. This supports that there were no MAX30001 failure events. Therefore all suspected degraded data is assumed to stem from other environmental factors (i.e., electrode disconnect or dynamic movement events). To summarize the self-test resistances, the average and standard deviation of 8 kHz, 18 kHz, 40 kHz, 80 kHz, and 128 kHz measurements for both days across all participants along with an in-lab comparison case are given in Figure 5. The in-lab comparison data was collected from a sensor system operational in a fixed setting (motionless at a workbench). Each of the sensing systems had similar average self-test values for each frequency, shown in Figures 5A,B, but there was a larger range in standard deviations observed. While each case has standard deviations < 0.15 Ω, this does highlight that variations between assembled systems and operation in different conditions (activity, temperature, motion) do impact the range of reported values. This is important for interpretation of data in free-living conditions, especially for detecting mΩ changes in tissue impedance. Designers should consider how the precision of their measurements may be impacted from free-living conditions that may not have been emulated during in-lab testing of precision and accuracy.

### 3.3 Bioimpedance Data Quality Classification

Assessing data quality is a critical step for all systems to ensure errors in equipment or measurement conditions are handled appropriately, otherwise data artifacts can be introduced during processing and interpretation. This is especially important for the wearable system and data presented in this work which was collected in an unsupervised environment. BIS data collected in a free-living environment can be subject to various sources of error including motion artifacts, electrode disconnect events, electrode aging, cabling damage/disconnects, and electronics/sensor damage. While sensor damage was eliminated as a source of error using the on-board BIS self-testing, the other identified error types could have impacted data quality. While events that impact data quality can be captured during direct supervision in a clinical or lab environment (with new measurements collected after correction or elimination of the error source), this was not possible during this unsupervised free-living pilot. Therefore, in post-processing it is critical to identify, report, and correct or remove data artifacts in bioimpedance datasets collected using wearable systems.

One method to identify degraded data includes threshold and trend analysis (Freeborn and Critcher, 2021). This method identifies impedance values that exceed thresholds established using literature reports of localized tissue values and/or violate the decreasing resistance with increasing frequency trend expected of biological tissues. For establishing an initial threshold range, existing studies that have collected localized bioimpedance using tetrapolar configurations from human subjects have reported resistances of:

- Approximately 70–100 Ω for 50 kHz resistances of lower and upper limb muscle groups of young and older adults reported (Kortman et al., 2013);
- Approximately 37–68 Ω for 50 kHz resistances of lower limbs of injured and non-injured football players (Nescolarde et al., 2013);
- Approximately 27–46 Ω in the range from 10 to 100 kHz in studies of localized bicep tissues before and after fatiguing activity (Fu and Freeborn, 2018);
- 45.87 ± 12.77 Ω and 46.26 ± 13.71 Ω for 50 kHz resistances of the right and left (transversal) thigh during test-retest studies (Honorato et al., 2021).

The values of resistance across these studies are within the range 10–200 Ω for frequencies from 1 kHz to 1 MHz. This can be exploited to set an expected threshold within which localized bioimpedance should fall. Measurements beyond this range are expected to be data artifacts. In addition to resistance thresholds, the bioimpedance phase angle $\left(Z_{PA}=\tan^{1}\;(X∕R)\right)$ is expected to be within the $-15^{{}^{\circ}}<Z_{PA}<-1^{{}^{\circ}}$, with values that approach −90° indicating measurement of an ideal capacitance and values greater than 0° indicating an inductive measurement. Both of those cases are not representative of a biological tissue and can be excluded.

Another feature that can be utilized to identify potential data artifacts is the frequency-dependent impedance of tissues. Experimental data exhibits a trend of decreasing resistance with increasing measurement frequency. As a result, for a single sweep of N resistance measurements at increasing frequencies where $f_{1}<f_{2}<\ldots <f_{N}$, it is expected that:

(1) $$R\;(f_{1})>R\;(f_{2})>\ldots >R\;(f_{N-1})>R\;(f_{N})$$

This decreasing resistance trend can identify potential data artifacts in a single sweep of multi-frequency measurements collected over a short-time interval (which assumes the “state” of the tissue is constant over this period).

Based on this, resistances such that $R(f_{i+1})>R(f_{i})$, where 1<i<N, could indicate measurements have degraded quality from violations that the “state” of the tissue is constant. An event that could cause this violation would be a muscle contraction which has been shown to increase tissue resistance (Li et al., 2016; Kitchin and Freeborn, 2019). Depending on the length of contraction, this increase in resistance could impact one or more of the discrete frequency measurements. Alternatively, it could be a sign that the tissue may have come under compression by an external force during free living (e.g., bumping into an object).

In a previous study of a single-participant knee tissue dataset 0.04 and 3.50 % of data was identified as being threshold and trend artifacts, respectively (Freeborn and Critcher, 2021). However, a significant limitation of that effort was only data from a single participant was analyzed. Here the resistance, phase thresholds, trend analysis was applied to the collected 8–128 kHz impedance data to evaluate if the previously reported level of BIS artifacts was consistent across additional participants and longer data collection period. The specific thresholds applied here were:

(2) $$10\Omega <R\;(f_{\;8kHz-128kHz})<200\Omega$$

(3) $$-15^{{}_{{}^{\circ}}}<\tan^{-1}\left(\frac{X(f_{8\text{kHz}-128\text{kHz}})}{R(f_{8\text{kHz}-128\text{kHz}})}\right)<-1^{{}_{{}^{\circ}}}$$

(4) $$R\;(f_{8kHz})>R\;(f_{18kHz})>\ldots >R\;(f_{128kHz})$$

All individual datapoints that violated one of Eq. 2 to Eq. 4 were labelled as potential data artifacts. As a conservative first approach, all resistance and reactance datapoints in a multi-frequency sweep with at least 1 potential data artifact were also classified as artifacts.

**FIGURE 6 ∣:**
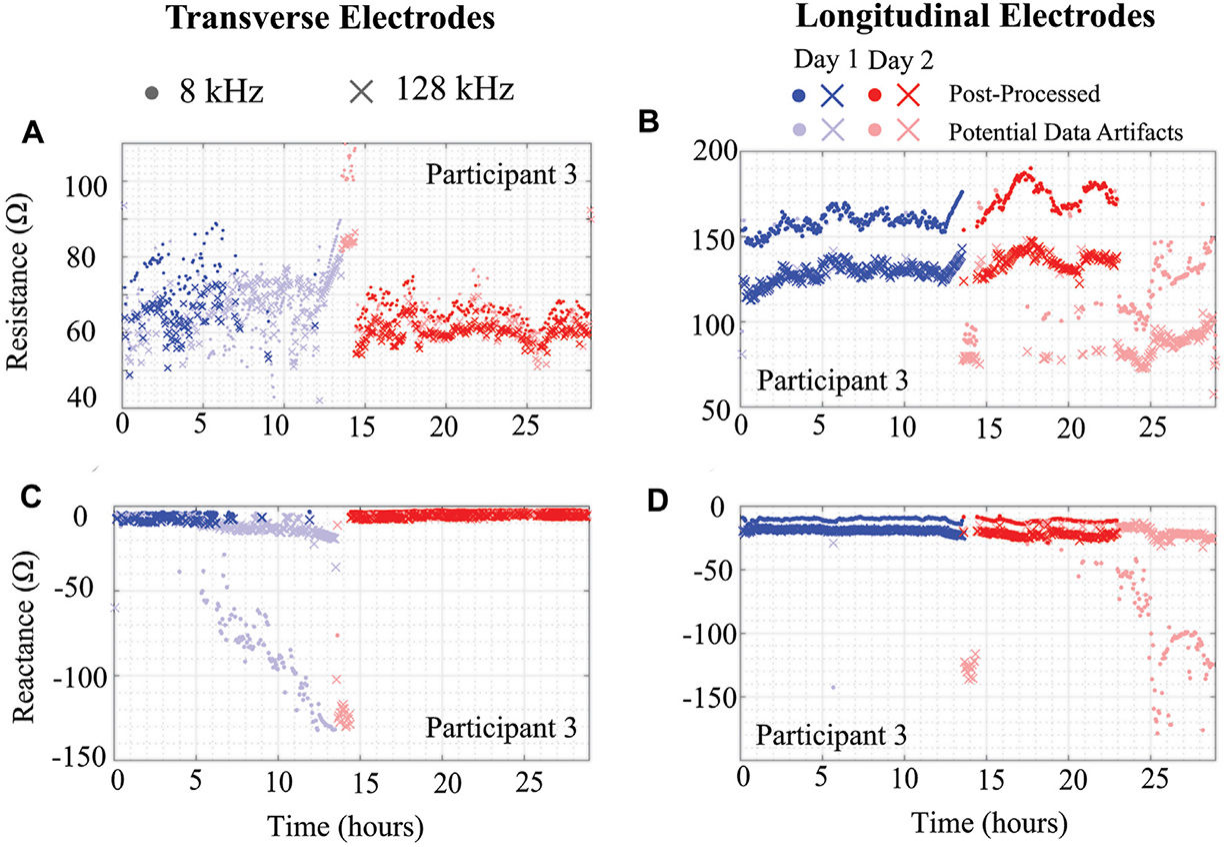
**(A,C)** Transverse and **(B,D)** longitudinal knee tissue resistance and reactance (8 kHz, 128 kHz) of Participant 3 using wearable sensing system over 2 days.

To illustrate the importance of data quality assessment applied to free-living BIS data, data from participant 3 across the 2 days is shown in Figure 6. In this figure, the 8 kHz (circle) and 128 kHz (x symbol) data with lighter shades represents data artifact (identified using threshold and trend detection) with primal color representing the specific day. Figures 6A-D plot the resistance and reactance of transverse and longitudinal electrode locations, respectively. Observing Figures 6A,C, note that over half the day one transverse electrode data was identified as data artifacts. The impact of using data with and without artifacts to generate descriptive statistics of tissue impedance is quantified in Table 2. This table presents the mean 8 kHz resistance and reactance using both datasets. The most significant resistance difference between pre- and post-processed data was 27.7 Ω (Day 2 - Longitudinal), with differences in reactance reaching 114.3 Ω (Day 2 - Longitudinal). Previously reported localized tissue alterations resulting from exercise and activity were < 10 Ω Fu and Freeborn (2018); Freeborn et al. (2020), which could not be accurately identified if data artifacts are not identified and removed from analysis and interpretation.

**FIGURE 7 ∣:**
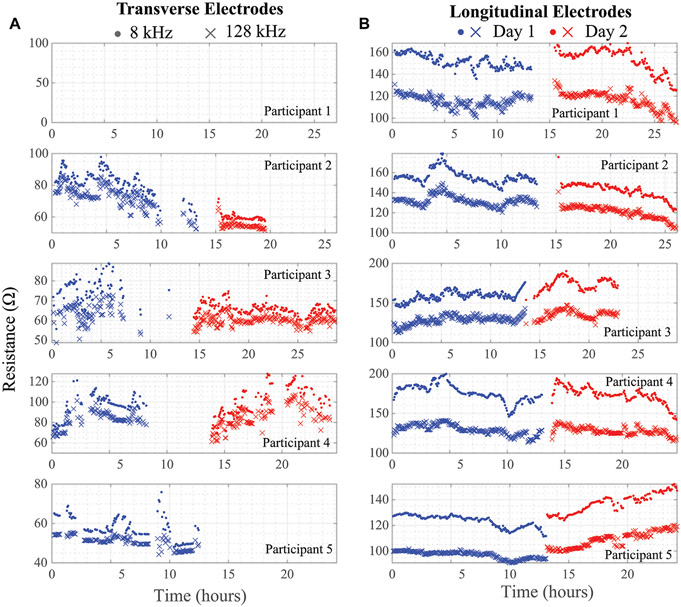
**(A)** Transverse and **(B)** longitudinal knee tissue resistance (8 kHz, 128 kHz) collected during free-living across 2 days from 5 healthy adult participants using wearable sensing system.

**FIGURE 8 ∣:**
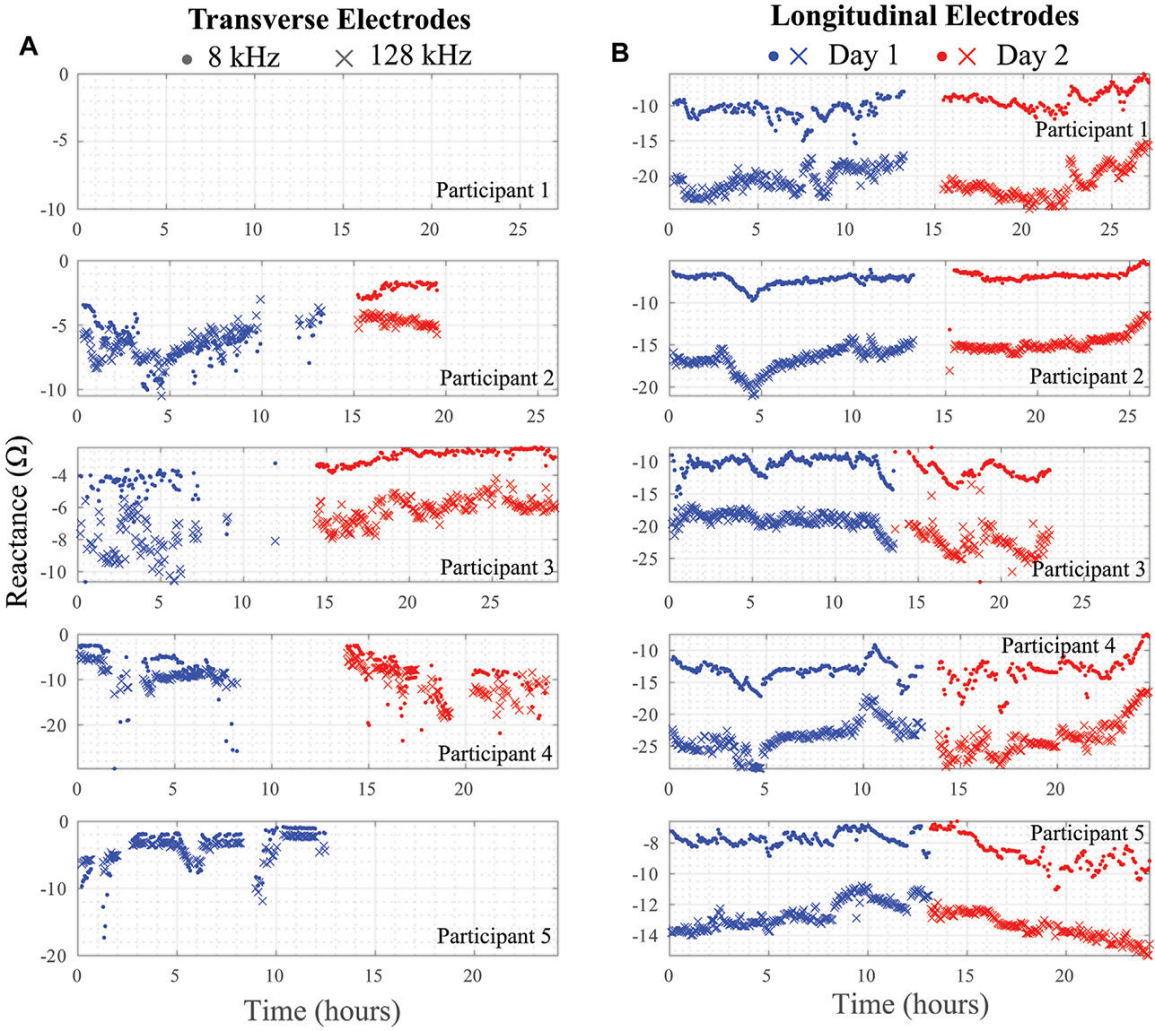
**(A)** Transverse and **(B)** longitudinal knee tissue reactance (8 kHz, 128 kHz) collected during free-living across 2 days from 5 healthy adult participants using wearable sensing system.

Using the threshold and trend identification methods, the percentage of artifact-free data in the total data from each participant is shown in Table 3 broken out per day and per electrode configuration. The artifact-free percentage was calculated by taking the ratio of datapoints that remained after the threshold/trend processes were applied and the total number of datapoints for that entire day. The processed resistance and reactance (at 8 and 128 kHz) after removal of the potential data artifacts are given in Figures 7, 8, respectively.

Reviewing the percentages in Table 3 supports that the longitudinal electrode configuration has a greater percentage of artifact free data (ranging from 47.4 to 96.4% with an average of 84.5%) than the transverse configuration (ranging from 0.0 to 71.8% with an average of 35.9%). This significant difference is attributed to differences in location of the electrodes. Greater movement is expected of the transverse electrode configuration leading to more significant geometry alterations of knee site tissue and shifting of the brace/electrodes that degrades data quality and increases the number of data artifacts.

Note that resistance/reactance data for Participant 1 is not presented in Figures 7, 8 for the transverse electrode configuration, indicating that all collected data was an artifact using the post-processing cleaning. In support that the post-processing cleaning is appropriate, a cabling problem was identified after the return of the wearable from Participant 1 to the study personnel that prevented data collection. Therefore, the threshold and trend analysis correctly identified all the artifacts in this test case supporting that it is accurate and appropriate data cleaning with this wearable.

### 3.4 Inter-Day Comparisons

On each day of data collection, the participants were required to replace adhesive electrodes in the brace and appropriately position the brace so the electrodes contacted the tissue at the sites of interest. This introduces variations in electrode positioning each day that the brace is worn. To assess the variability as a result of this approach, the average (and standard deviation) 8 and 128 kHz resistance and reactance for the longitudinal electrode configurations were calculated using the initial 20 artifact-free measurements from each participant for both days. These values are detailed in Table 4. The averages are limited to the initial 20 measurements (representing approximately 1 h of time) because it is expected that participants will be in a similar physiological state in this period for both days. Comparing the values given in Table 4 the differences between days for the longitudinal resistances ranges from 0.04 to 7.40% (2.20% average) and 0.01–32.8% (11.01% average) for reactances. This small difference of the average resistance between days supports that participants were able to position the brace appropriately (for longitudinal configurations). This also highlights the use of this commercial knee-brace as a possible solution to achieving repeatable electrode placement. The significant number of data artifacts in the transverse configurations prevents exploring the inter-day bioimpedance values of this site.

### 3.5 Environmental Conditions and Motion Context

Beyond BIS data the wearable system captured environmental (temperature, humidity) and motion (3-axis acceleration) data during the 2-days collection period. This data is intended to inform and quantify the context in which the BIS data was collected and conditions that could impact interpretation. For example, periods of high activity could impact data quality by decreasing contact quality between electrodes and surface tissues, increased sweat during activity could alter electrode adhesion, sudden changes in body position after extended periods of inactivity could cause significant fluid shifts and impact tissue bioimpedance.

**FIGURE 9 ∣:**
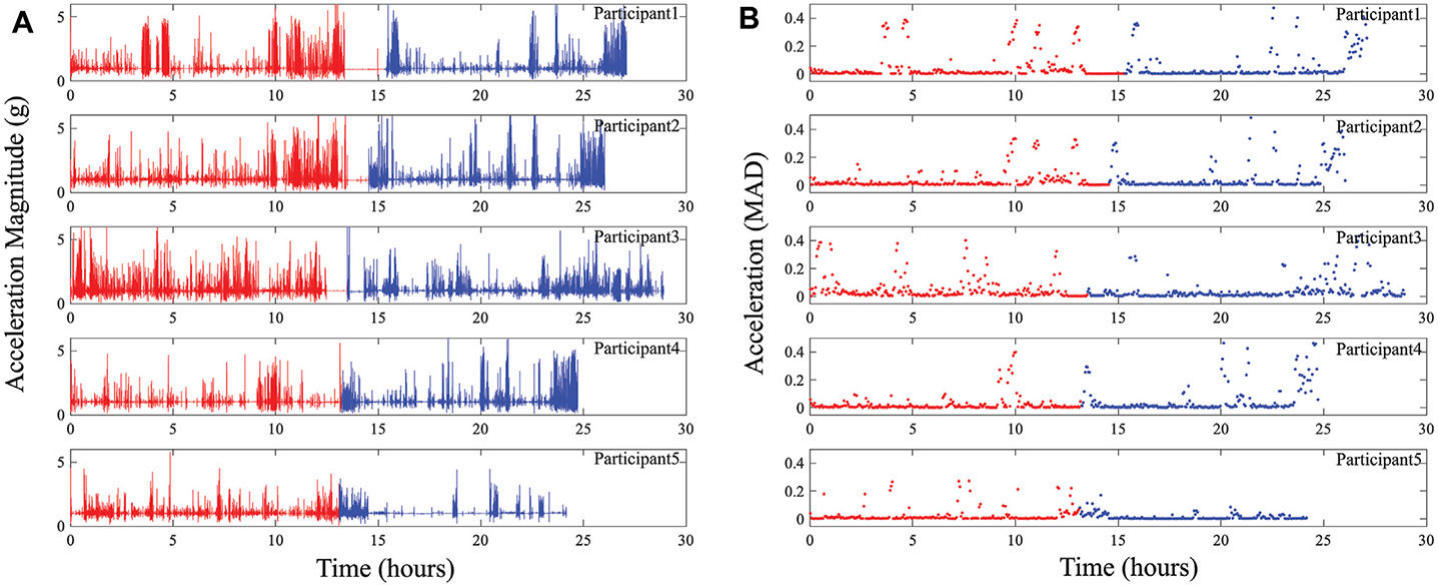
**(A)** Acceleration magnitude and **(B)** MAD representing activity at participants knee collected using wearable system across days 1 (red) and 2 (blue) of data collection.

To highlight the knee activity of participants wearing the brace in this pilot study, the acceleration magnitude $\left(|a_{i}|\right)$ and mean absolute difference (MAD) were generated using the 3-axis accelerometer data to reduce dimensionality. The metrics were calculated using:

(5) $$|\bar{a}|=\frac{1}{N}\sum_{i=1}^{N}|a_{i}|$$

(6) $$\text{MAD}=\frac{1}{N}\sum_{i=1}^{N}|a_{i}-\bar{a}|$$

where $x_{i}$, $y_{i}$, $z_{i}$ represents the x, y, and z-axis accelerations (in g’s) at a single time instant $\left(t_{i}\right)$, and N represents the total number of acceleration datapoints. The use of (5) reduces the 3-axis data to a single value at each time instant representative of the total overall acceleration with the MAD value generating a single value per measurement period (with higher values indicating higher accelerations and periods of extended movement). The magnitude and MAD values were calculated using 21,792 acceleration datapoints which represents the total dataset collected at 200 Hz between bioimpedance measurements in the system sequence (described earlier in Section 2.3). The generated magnitude and MAD for all five participants across both days of data collection are given in Figure 9. Similar to the BIS data, the acceleration values show a high-level of variability. This is a result of the participant activities, which while unsupervised, involved significant periods of sitting (for computer focused work) with brief periods of indoor and outdoor walking. The brief periods of inactivity are represented most clearly in Figure 9B, with MAD < 0.05 attributed to periods of sitting.

**FIGURE 10 ∣:**
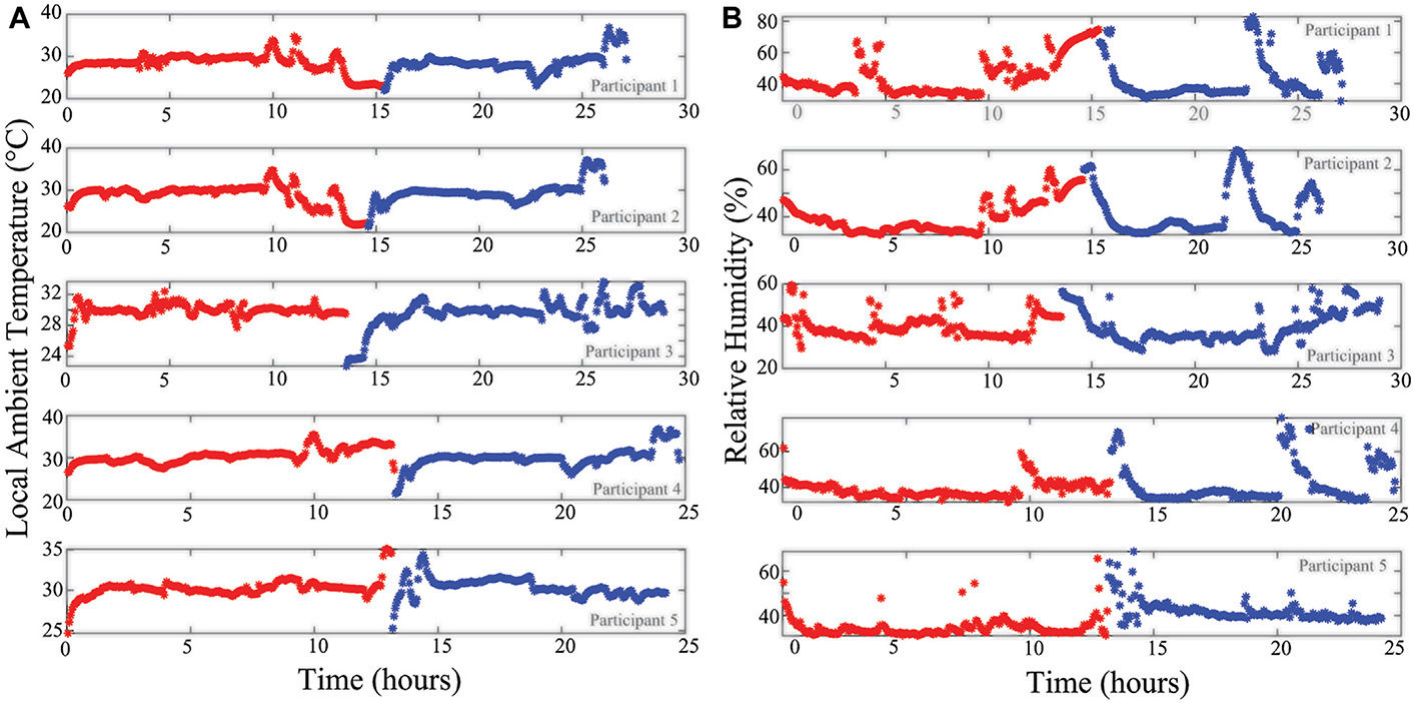
**(A)** Temperature and **(B)** relative humidity of local wearable environment across day 1 (red) and 2 (blue) of participant data collection.

**FIGURE 11 ∣:**
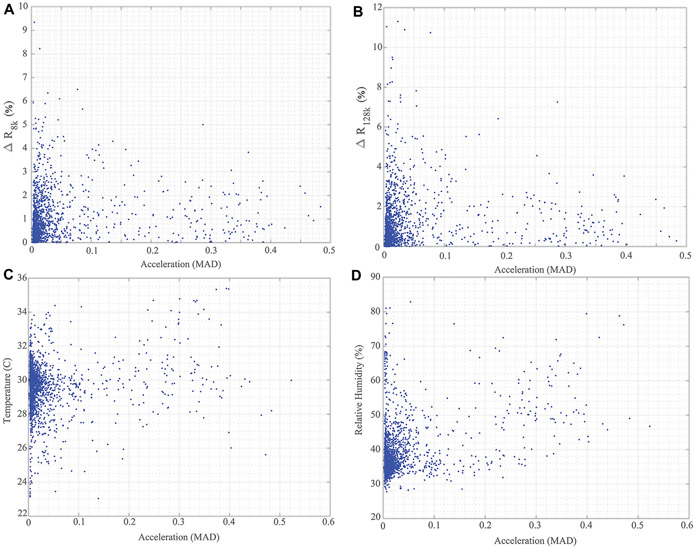
Comparisons of **(A)** $\Delta R_{8k}$, **(B)** $\Delta R_{128Kk}$ for longitudinal knee bioimpedance, **(C)** temperature, and **(D)** humidity reported by wearable with acceleration MAD from participants across 2-day data collection.

Additionally, the localized temperature and humidity data from all 5 study participants are given in Figures 10A,B, respectively. This provides an overview of the local conditions inside the brace as the system PCB is enclosed in a fabric pocket fastened to the brace exterior. While the temperature/humidity data does vary throughout the data collection period for all participants, it provides the opportunity to evaluate the strength of associations between the different data types collected. It also allows for further investigation and use in data cleaning/interpretation algorithms. As an example, Figure 11 presents comparisons of the 8 and 128 kHz resistance differences of longitudinal knee bioimpedance, temperature, and humidity with acceleration MAD. For reference, the 8 and 128 kHz resistance differences were calculated as:

(7) $$\Delta R_{8k_{i}}=\frac{|R_{8k_{i}}-R_{8k_{i-1}}|}{R_{8k_{i-1}}}\times 100$$

(8) $$\Delta R_{128k_{i}}=\frac{|R_{128k_{i}}-R_{128k_{i-1}}|}{R_{128k_{i-1}}}\times 100$$

where $R_{x_{i}}$ and $R_{x_{i-1}}$ represent two consecutive resistance measurements collected from multi-frequency sweeps that did not have any identified data artifacts (as discussed in Section 3.3). Only longitudinal measurements were included in this analysis because of the high number of data artifacts identified in the transverse datasets.

It is hypothesized that knee movement/activity will be correlated with changes in knee tissue bioimpedance (resulting from contraction events and fluid shifts resulting from activity), brace temperature (resulting from heat transfer to the brace through skin contact), and brace relative humidity (resulting from increases of sweat during periods of activity). A Pearson’s product-moment correlation was run to assess these relationships using the data generated from the 5 study participants. From this analysis, the following statistically significant correlations were reported:

- a small positive correlation (*r* (1920) = 0.153, *p* < 0.05) between $\Delta R_{8k}$ and MAD;
- a small positive correlation (*r* (1920) = 0.1132, *p* < 0.05) between $\Delta R_{128k}$ and MAD;
- a small positive correlation (*r* (2058) = 0.12, *p* < 0.05) between brace temperature and MAD; and
- a moderate positive correlation (*r* (2058) = 0.423, *p* < 0.05) between brace relative humidity and MAD.

These correlations support the hypotheses regarding knee movement/activity and tissue impedance/local brace conditions though further investigations are necessary to validate and utilize these associations for data interpretation.

## 4. USER FEEDBACK

While this preliminary pilot only required participants to use the device for 2 days, the long-term aim is to facilitate continuous monitoring of the localized knee spanning days and weeks to evaluate trends over large timescales for applications related to disease progression or identification of acute injury. Long-term adoption requires that this system not only meet technical requirements for accurate and reliable data collection but that users can integrate this device into their lives and are comfortable with long-term wear (especially because it directly contacts the tissue). To collect information about the participants experiences with the brace (to inform future revisions) each participant completed a post-study questionnaire with a series of Likert-scale and open-ended questions. For each question, the available responses included strongly disagree (−2), disagree (−1), neutral (0), agree (1), strongly agree (2), and N/A with positive numbers related to a level of agreeance and negative numbers a level of disagreeance. The specific Likert-scale questions (along with the average score) are below:

- The training that I received on the brace provided all the details I needed. (2.0)
- I had to talk to the study team for help with the brace. (−1.2)
- The fabric was comfortable against my skin. (−0.2)
- The electrodes were comfortable against my skin. (−1.0)
- The electrodes caused a rash on my skin. (−0.6)
- I was able to walk around and do my normal activities while wearing the brace. (1.0)
- I was worried about bumping the brace and the sensors in it. (0.4)
- I did bump the brace and was worried that I damaged it. (−1.67)
- I sometimes forgot I was wearing the brace. (0.2)
- I really wanted to take the brace off each day. (1.0)

In terms of training, participants agreed that the training they received was adequate and did not feel they had to contact the study team for help. This supports that participants felt confident in setting up electrodes in the brace, placing the brace appropriately on the knee, and recharging the system. In terms of using the brace, participants agreed daily activities could be completed while wearing the brace without feelings that they had to be careful about damaging the brace. However, in terms of comfort participants did not feel the adhesive electrodes were comfortable against their skin and had strong agreement/desire to take the brace off at the end of each day. This indicates that this wearable needs revisions in terms of how the system interfaces to the tissue for data collection (e.g., dry electrodes over wet adhesive electrodes) and the selection of fabric/fastening for improved comfort.

In the collected responses to the open-ended questions, which asked for additional comments and/or recommendations about the brace and how to better the fit or make it more comfortable, participant responses included:

- Feedback on the use of the wet Ag/AgCl electrodes: “It would be fantastic if you could find a way to make the gel less messy”, “The electrodes peel off/on while moving.. ..if there’s a way to keep the electrodes more flush with the skin so the rubbing/peeling doesn’t happen, that would be amazing.”
- Feedback on available sizing of the brace: “Knee section felt a little loose but the other parts were tight”, “Variable sizing for the brace would help the brace fit comfortably on more unique body types.”

This feedback complements responses received to the Likert-scale questions, providing further insight on possible revisions for the brace to improve comfort/usability, with clear trends emerging that efforts should focus on improving the tissue/electrode interface for comfort and improving the fit of the individual brace to each specific participant.

## 5 DISCUSSION

This study assessed the performance of a knee-focused wearable sensing system by collecting both quantitative sensor data and qualitative user-experiences. These data provide insights regarding the reliability of data (i.e., quantity of artifact free data) and limitations of this design in unsupervised free-living environments. While the system in this effort is not the first wearable BIS system, with a range of wearable BIS systems presented in the literature (Hersek et al., 2016; Rossi et al., 2017; Teague et al., 2020; Ngo et al., 2022; Sanchez-Perez et al., 2022), it does advance the use BIS wearables beyond controlled laboratory or clinical settings. For a comprehensive comparison of existing BIS wearables to this system, details of existing systems electrode placements, on-body locations, length of data collection, location of use, number of participants, and processing methods applied to collected BIS data are provided in Table 5.

This work is the only system utilizing a commercially available form-factor (off the shelf knee brace) with electrodes integrated into the wearable textile. Most other BIS wearable designs (except for (Rossi et al., 2017)) require the electrodes to be manually placed independent of the wearable textile. The intent of this approach was to reduce the effort by users to easily and consistently place electrodes. The user feedback on feeling confident in using the brace and the small variation in longitudinal data comparing measurements within the first hour of use support that this approach is effective in terms of both usability and consistency. In comparison to other wearable BIS systems, this work is the only study that has collected near-continuous sensor data in an unsupervised free-living environment with most other studies collected data in a lab or clinic setting and for only short duration measurements. Finally, this effort advances methods to identify data artifacts in BIS data collected during free-living towards removing degraded data from impacting data interpretation. While Sanchez-Perez et al. have also presented a method for quality assessment of BIS data for heart failure related applications (Sanchez-Perez et al., 2022), this was limited to controlled in-lab and clinical data.

In terms of performance, the multi-site BIS functionality advances capabilities of a knee monitoring system to assess multiple localized tissue sites without degrading performance due to the additional electronics. Further, this functionality enables on-board self-testing to increase operational confidence in uncontrolled conditions which is increasingly important as designs transition from the laboratory to free-living.

The post-processing analysis utilizing resistance and phase thresholds as well as multi-frequency trend analysis were successful at identifying data artifacts in the free-living data generated by the study participants. The successful identification of 100% of the data during a cable disconnect event (i.e. Participant 1 transverse data) as data-artifacts supports this approach. There are further opportunities to improve artifact identification methods, which could integrate multi-modal data such as motion sensing and improving knowledge of localized impedance alterations from activity. While there are details of impedance alterations due to injury (Nescolarde et al., 2013), fatigue (Freeborn et al., 2020), and fluid shifts (Fenech and Jaffrin, 2004) these are not from localized knee tissues. This limits the confidence in estimating what magnitude of alterations could occur with short-term alterations that exceed these levels of change being identified as data-artifacts. Therefore, further research into the magnitude of knee tissue bioimpedance alterations for various free-living activities/events is recommended.

It is also clear from the processed impedance data presented in Figures 7, 8 that choice of electrode locations had a significant impact on data-quality. The longitudinal configuration had approximately 84.5% artifact-free data compared to 35.9% for the transverse configuration (which limited further analysis of the transverse bioimpedance data). The longitudinal configuration placed electrodes on tissue locations with limited movement (see Figure 1C) and benefited from placement under the fastening straps of the brace (see Figure 1B). Both of which improved the contact quality of the electrodes/tissues. The source of data artifacts for the transverse electrodes is attributed to dynamic activity which impacts tissue geometry, electrode contact quality, and brace fit. While improvements in fit are possible by transitioning to personalized sizing/tighter fabrics, electrodes fixed to the brace will continue to be effected by fabric movement during activity or specific knee positioning (e.g., bent during sitting) that exert force on the electrodes and reduce contact quality with the skin surface. This highlights the trade-offs between choice of bracing and tissue/electrode interfacing which should be investigated further to identify an optimum solution to improve collection reliability.

While previous wearable knee systems have used accelerometer data to classify body positions Hersek et al. (2016) the collection of multi-modal data (e.g., bioimpedance and acceleration) provides the opportunity to explore how movement (intensity, duration, type) impacts localized knee tissues. As an example, the preliminary data reported here supports that there is a small association between the short-term level of knee activity and alterations in longitudinal knee tissue resistance. However, this analysis has not factored in length of activity or body position. Both factors are expected to impact tissue bioimpedance and should be considered in future studies to understand the time-course alterations in knee tissues across all types of free-living activities.

The experiences of the participants offered insights in brace usability focused on fit and comfort, with feedback that adhesive electrodes were not comfortable against participants skin with each expressing a strong desire to take the brace off at the end of each day. While this brace was adequate in usability for a short-term pilot study it is not expected to meet participant expectations for a daily use wearable. In terms of improvements, transitioning to dry electrodes using conductive fabric (Wang et al., 2019), embroidery (Logothetis et al., 2019), or bare metal (Kusche et al., 2018) is expected to improve the comfort and reduce the setup required for daily use. However, this may also increase data artifacts as a result of poor contact quality due to the loss of adhesive and electrolytic solution. This highlights why multi-model data is also important for future designs, the use of humidity data local to the brace and textiles could identify the sweat in the brace that could effect contact quality and collected data. In terms of repeatability, the between day differences of the 8 and 128 kHz longitudinal resistances and reactances averaged to 2.20 and 11.01%, respectively. For comparison, recent 50 kHz test-retest measurements of thigh tissue resistance had absolute differences less than 5.5 and 6.26% for resistance and reactance, respectively, collected in a controlled clinical setting (Honorato et al., 2021). This supports the participants self-setup of the knee brace with installed electrodes achieved similar levels of repeatability as the clinical placements supporting the continued use of this method.

It is also important to note that the views regarding comfort and usability are limited because of sampling only healthy young adults. While the fit/comfort experiences are expected to translate to all populations, the confidence in setup and impact on daily activities may not be transferable to populations of older adults and adults with knee injuries or disease. Therefore further usability testing with a wider sampling of adults across the age-span and including adults with and without knee pathologies (e.g. osteoarthritis of the knee and other joints) is recommended to understand the further needs of these populations and their experiences with wearable technologies for knee monitoring.

## 6 CONCLUSION

This pilot study demonstrated the performance of a wearable sensing system for knee monitoring through its use by 5 healthy participants across 2 days of unsupervised free-living. The pilot data demonstrated the variability in tissue impedance during free-living and the need to identify and clean data artifacts to reduce their impact on data interpretation, which is especially important to transition designs from lab/clinical environments to free-living. An artifact identification process (threshold, trend, and phase methods) is presented that suggests an average of 84.5 and 35.9% artifact free-data was collected from longitudinal and transverse electrode configurations (with higher artifacts resulting from on-body locations with greater movement). Using the collected multi-model sensor data, small to moderate positive correlations were reported between impedance and contextual data with respect to acceleration data, supporting the utility of this wearable system to identify associations between knee measurement context and localized tissue impedance to gain greater insight into the tissue and sources of change. Positive user feedback and consistency between BIS measurements supports that users are confident and capable in using this system after training. But improvements in comfort, sizing, and tissue interfacing are needed to reduce obtrusiveness and increase adoption of this system for future studies.

## Figures and Tables

**TABLE 1 ∣:** Participant knee measurements.

Part	M1 (in.)	M2 (in.)	M3 (in.)	Brace size
1	15	13.75	12.75	S/M
2	16.5	15	14	L
3	17	14.5	14	XL
4	18	15.75	14	3XL
5	17.5	16	14	XL
**Average**	16.8	15	13.75	-

**TABLE 2 ∣:** Day 1 and 2 mean 8 kHz resistance and reactance of Participant 3 using pre- and post-processed data.

Dataset	Transverse		Longitudinal	
	Day 1	Day 2	Day 1	Day 2
8 kHz Resistance (Ω)				
Pre-	69.80	68.12	157.57	146.23
Post-	74.71	65.71	157.58	173.90
8 kHz Reactance (Ω)				
	Day 1	Day 2	Day 1	Day 2
Pre-	−59.75	−70.94	−23.21	−126.03
Post-	−4.58	−2.82	−10.37	−11.76

**TABLE 3 ∣:** Percentage of transverse and longitudinal knee tissue bioimpedance classified as high-quality (e.g., not degraded) using threshold and trend

Participant	Transverse		Longitudinal	
	Day 1 (%)	Day 2 (%)	Day 1 (%)	Day 2 (%)
1	0.0	0.0	76.8	88.2
2	59.4	34.3	88.2	90.3
3	35.3	71.8	96.4	47.4
4	42.5	56.0	89.1	88.0
5	59.6	0.0	91.8	88.5
**Average**		35.9		84.5

**TABLE 4 ∣:** Average 8 and 128 kHz resistance/reactance from longitudinal electrodes generated using first 20 artifact-free measurements.

Participant	$R_{8{\mathrm{kH}}_{z}}\;(\Omega )$		$R_{128{\mathrm{kH}}_{z}}\;(\Omega )$		$X_{8{\mathrm{kH}}_{z}}\;(\Omega )$		$X_{128{\mathrm{kH}}_{z}}\;(\Omega )$	
	Day1	Day2	Day1	Day2	Day1	Day2	Day1	Day2
1	159.1	160.8	123.4	126.2	−9.7	−8.8	−21.2	−21.2
	±1.47	±3.79	±2.59	±4.41	±0.5	±0.24	±1.10	±0.52
2	155.9	154.6	132.9	130.1	−6.8	−7.9	−16.9	−15.9
	±1.02	±13.98	±0.53	±7.41	±0.18	±3.50	±0.37	±1.44
3	148.3	156.3	117.0	125.6	−12.7	−8.5	−19.3	−19.9
	±3.20	±2.47	±3.64	±1.89	±2.16	±0.12	±0.97	±0.46
4	178.7	178.8	130.2	128.8	−12.2	−14.7	−24.5	−25.4
	±4.61	±9.40	±3.10	±6.27	±0.65	±4.06	±0.86	±1.46
5	128.4	127.5	100.2	101.3	−7.7	−7.0	−13.7	−12.5
	±0.61	±0.84	±0.18	±1.07	±0.25	±0.12	±0.11	±0.28

**TABLE 5 ∣:** Wearable bioimpedance system comparisons from the literature.

Study	Description	Electrode placement	On-Body location	Data collection length	Deployed	Num. Of part	Processing
Hersek et al. (2016)	Two bands above and below knee	Manual placement	Knee	60 s	Lab	49	Body position algorithm
Hersek et al. (2016)	Two bands above and below knee	Manual placement	Knee	Over 3 days, but not continuous	Lab	5	Body position algorithm
Rossi et al. (2017)	Patch	Manual placement, integrated into patch	Chest	^a^ND	Lab	1	ND
Teague et al. (2020)	Two bands above and below knee	Manual placement with stencil	Knee	10 cycle of exercises	In-lab	1	Averaging
Ngo et al. (2022)	ND	Manual Placement	Thigh	ND	In-lab	1	ND
Sanchez-Perez et al. (2022)	Arm band	Manual Placement	Chest	ND	Lab	10	Filtering and quality assessment
Sanchez-Perez et al. (2022)	Arm band	Manual Placement	Chest	3 meas. over 36 h	Clinical setting	14	Filtering and quality assessment
This work	Commercial knee brace	Integrated	Knee	2-days continuous	Free-living	5	Quality assessment

a ND, not discussed.

## Data Availability

The raw data supporting the conclusion of this article will be made available by the authors, without undue reservation.
